# Glucose‐Lowering Properties of a Tea Formulation Containing *Citrullus lanatus* Peel, *Ananas comosus* Peel, and *Mentha spicata* L. Leaf Extracts in Dexamethasone‐Induced Insulin‐Resistant Wistar Rats

**DOI:** 10.1002/fsn3.71368

**Published:** 2025-12-30

**Authors:** Irwin Leoda Begnone, Laurette Blandine Mezajoug Kenfack, Rosane Matsinkou Soh

**Affiliations:** ^1^ Department of Food Science and Nutrition, National School of Agro‐Industrial Sciences The University of Ngaoundere Ngaoundere Cameroon; ^2^ Department of Food Engineering and Quality Control of the University Institute of Technology (IUT) University of Ngaoundere Ngaoundere Cameroon

**Keywords:** *Citrilus lanatus*, glucose‐lowering, insulin resistance, *Mentha spicata*, *Pineapple comosus*, tea

## Abstract

This study investigated the glucose‐lowering properties of a tea blend combining *Citrullus lanatus* , *Ananas comosus* , and *Mentha spicata* . To achieve this, we initially characterized the raw materials and subsequently developed a mixture design, resulting in 11 distinct tea formulations. Physicochemical analyses of the various tea formulations enabled the development of mathematical models describing the relationships between the characteristics and raw material quantities. Using Design Expert 13 software, optimization was performed to maximize antioxidant content, facilitated by a specification sheet. After microbiological analysis, the optimal tea formulation was administered to male Wistar rats at doses of 300 and 500 mg/kg body weight during hypoglycemia, oral glucose tolerance, and insulin resistance tests induced by dexamethasone. Upon completion of the 10‐day experiment, the diabetic animals were euthanized, and biochemical parameters were evaluated. The optimized antioxidant‐rich tea formulation contained flavonoids (813.986 mg/mL), tannins (0.056 mg/mL), polyphenols (2019.35 mg/mL), and vitamin C (0.735 mg/mL) and exhibited high antioxidant activity (128.86 mg/mL). Additionally, it met the required microbiological standards. The rat experiment indicated that both tea doses significantly (*p* < 0.05) improved glycemic control, comparable to the reference drug group. Additionally, the tea doses resulted in significant reductions in total cholesterol, triglycerides, and LDL cholesterol, as well as an increase in HDL cholesterol. Furthermore, the activity of intracellular enzymes ASAT and ALAT, as well as serum concentrations of urea, creatinine, and albumin, decreased significantly in response to the tea's action. The 500 mg/kg dose showed the highest efficacy, suggesting that it could be a potential alternative treatment for diabetic patients.

## Graphical abstract

The optimized tea blend exhibited high antioxidant activity and contained significant amounts of flavonoids, tannins, polyphenols, and vitamin C. In diabetic rats, the tea formulation significantly improved glycemic control, reduced lipid profiles, and enhanced liver and kidney function. The 500 mg/kg dose showed the highest efficacy, suggesting its potential as an alternative treatment for diabetes.

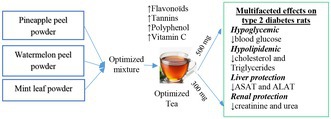

## 1 Introduction

Fruits have always been a staple in human diets, offering a rich mix of colors, flavors, and aromas. They provide a wealth of essential nutrients, particularly secondary metabolites, whether eaten fresh or processed. Industrial processing generates significant by‐products, including pulp, seeds, and peels, which can account for up to 52% of the fruit's weight (Cheok et al. 2018). These by‐products have a potential for reuse, as they contain valuable substances such as phenolic compounds, dietary fibers, and vitamins, consumed fresh or processed into various products (Messinese et al. 2024). Their by‐products, including peels, have been shown to possess anti‐diabetic properties (Ramya et al. 2020; Priya et al. 2024). These properties could be leveraged in the formulation of anti‐diabetic teas, offering a potential industrial application. An anti‐diabetic tea is an infusion of plants, usually tea or other botanicals, that contain bioactive compounds, which help regulate glucose metabolism, reduce diabetes complications, and improve insulin sensitivity (Ramya et al. 2020).

Diabetes is a metabolic disorder characterized by hyperglycemia, affecting over 537 million people worldwide (IDF 2021). The disease is divided into two main types: type 1, resulting from pancreatic problems, and type 2, resulting from insulin resistance (WHO 2019). Africa has the largest number of people unaware of their diabetes status, with an estimated 70% of diabetics remaining undiagnosed (Diawara et al. 2023). In Cameroon, diabetes affects over 615,000 people, with a higher prevalence in urban areas (IDF 2019). The disease constitutes a significant public health problem, requiring a multidisciplinary approach, including pharmacological agents, dietary recommendations, and daily routines. Recent years have seen extensive research into plants with anti‐diabetic properties, including *Citrullus lanatus* ( *C. lanatus* ) and *Ananas comosus* (*A. comosus*) peels, which have been found to possess therapeutic and pharmacological benefits (Sharma et al. 2024). Our goal was to boost the antioxidant potential of *C. lanatus* and *A. comosus* peels by blending them into a tea for diabetic patients. Given the potential taste and palatability issues with the peel mixture, we added *Mentha spicata* ( *M. spicata* ), a plant renowned for its therapeutic benefits, to enhance the tea's organoleptic properties. The tea obtained will then be tested for its hypoglycemic and anti‐hyperglycemic effects in normal and insulin resistant Wistar rats induced with dexamethasone.

## 2 Materials and Methods

### 2.1 Tea Production

#### 2.1.1 Production Process of Watermelon peel, Pineapple Peel, and Mint Leaf Powder

Once received at the laboratory, the fruit peels ( *C. lanatus* and *A. comosus* ) and mint leaves ( *M. spicata* ) were cleaned and washed. The watermelons (*Seminis apoorva* variety) were harvested from a farm in Ngaoundere, whereas the pineapples (*Pernambuco* variety) were purchased from a market in Yaounde, and the mint leaves were bought from the Dang market. After receipt, the raw materials were sorted, and infected fruits and defective mint leaves were discarded. The materials then underwent the following steps:

##### 2.1.1.1 Washing, Peeling, and Cutting

Fruits and leaves were washed three times with clean running water. Fruits were peeled using a stainless steel knife, and the peels were cut into small pieces (5–10 mm) and dried in a ventilated electric dryer.

##### 2.1.1.2 Withering

Watermelon and pineapple peels were withered at 25°C for 24 h in the shade, whereas mint leaves were withered for 8 h at 25°C.

##### 2.1.1.3 Rolling

Mint leaves were rolled for 30 min to deform and slightly break the cells, releasing enzymes and bioactive compounds.

##### 2.1.1.4 Drying

Watermelon and pineapple peels were dried at 50°C for 24 h, whereas mint leaves were dried at 35°C for 10 h.

##### 2.1.1.5 Weighing and Grinding

Peels and leaves were weighed and ground for 3 min using a hammer mill at 4000 rpm.

##### 2.1.1.6 Sieving

Powders were sieved at 400 μm to ensure optimal granulometry, eliminating coarse particles and ensuring better solubility and extraction of active compounds.

This process allows for the production of high‐quality powders from watermelon peels, pineapple peels, and mint leaves.

**FIGURE 1:**
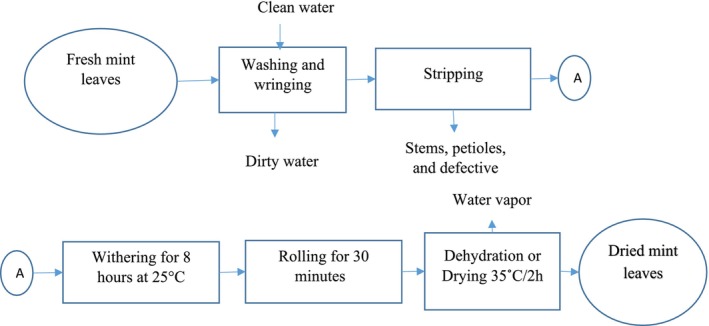
Method for obtaining mint leaves.

**FIGURE 2:**
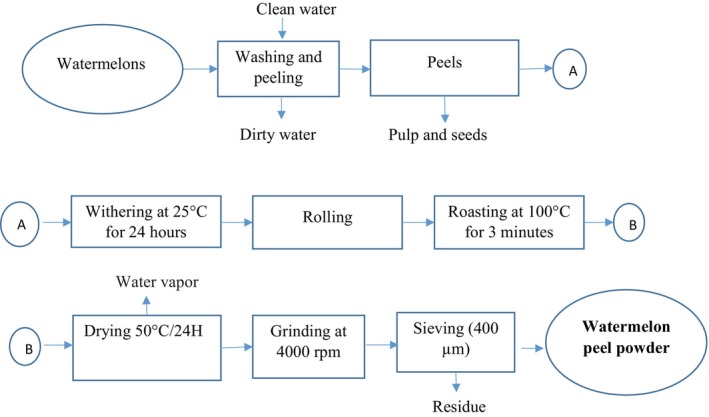
Watermelon rind powder production process.

**FIGURE 3:**
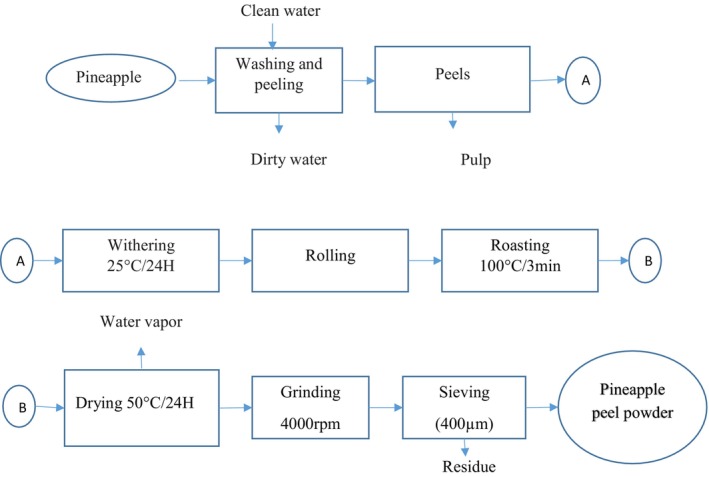
Process diagram for obtaining pineapple peel powder.

Figures 1, 2, 3 show the process flow diagrams for producing the various powders as previously described.

#### 2.1.2 Experimental Device for Obtaining the Mixture

The experimental design used here was a simplex‐centroid mixture design with constraints: *A. comosus* ≥ 50%, *C. lanatus* ≥ 40%, and *M. spicata* ≤ 20%. The objective was to enhance the antioxidant potential of our tea and improve its flavor and taste. However, we created a mixture design that would yield different formulations of our tea. The various raw materials obtained were then mixed to produce the tea.


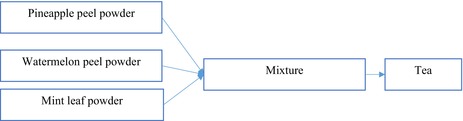


#### 2.1.3 Experimental Design

The factors selected for the mixture design, on the basis of literature review, are *A. comosus* , *C. lanatus* , and *M. spicata L*. The experimental domain is outlined in Table 1.

**TABLE 1:** Experimental domain of factors.

Factors	Low limit (%)	Upper limit (%)
*Ananas comosus*	40	50
*Citrillus lanatus*	35	45
*Mentha spicata* L	10	15

Design Expert software was used to generate a D‐Optimal mixture design consisting of 11 formulations, as outlined in Table 2.

**TABLE 2:** D‐optimal mixture design of 11 formulations.

Formulations	Ananas (%)	Pastèque (%)	Menthe (%)
F1	0.475	0.425	0.1
F2	0.5	0.35	0.15
F3	0.45	0.4	0.15
F4	0.43125	0.43125	0.1375
F5	0.4625	0.4125	0.125
F6	0.45	0.45	0.1
F7	0.5	0.4	0.1
F8	0.4	0.45	0.15
F9	0.4	0.45	0.15
F10	0.5	0.35	0.15
F11	0.5	0.375	0.125

### 2.2 Chemical Characterization of Raw Materials and Tea Formulations

The moisture and ash content of the samples were determined using the AOAC method (1990) by heating samples at 105°C to measure water content and incinerating samples at 550°C for 24 h to measure ash content (AOAC 1990). The soluble and total sugar content of the samples were determined using the DNS method described by Fischer and Stein (1961), which involves extraction and quantification of sugars on the basis of their reaction with DNS reagent to form an orange compound absorbing at 540 nm (Fischer and Stein 1961). Saponin content was determined using Koziol's method (1991), where 0.5 g of sample was mixed with distilled water, shaken, and the foam height was measured to calculate saponin concentration (Koziol 1991). The determination of certain bioactive compounds in the teas was carried out. The total polyphenol content was determined using the Folin–Ciocalteu method, where phenolic compounds were extracted with 70% ethanol and quantified spectrophotometrically on the basis of the reduction of Folin–Ciocalteu reagent to form a blue compound absorbing at 725 nm, with results expressed as gallic acid equivalents (Marigo and Gadal 1973; Dewanto et al. 2002; Wafa et al. 2014). The total flavonoid content was determined using the aluminum chloride (AlCl_3_) method, where flavonoids formed complexes with AlCl_3_ and were quantified spectrophotometrically at 430 nm, with results expressed as quercetin equivalents (Bahorun et al. 1996; Chang et al. 2002). Tannins were determined using the acidified vanillin method, where tannins react with vanillin to form a red‐colored complex absorbing at 500 nm, with results expressed as tannic acid equivalents (Bainbridge et al. 1996). The vitamin C content was determined using the titrimetric method of Harris and Ray (1935), where the reduction of 2,6‐dichlorophenol indophenol (2,6‐DCIP) by ascorbic acid was used to quantify vitamin C content, with extraction protocol adapted from Sun et al. (1998) (Harris and Ray 1935; Sun et al. 1998). The antioxidant activity was evaluated using the DPPH (2,2‐diphenyl‐1‐picrylhydrazyl) method, where the ability of the extracts to reduce DPPH was measured spectrophotometrically at 517 nm, with results expressed as IC_50_ values (Sánchez‐Moreno 2002; Athemena et al. 2010). Mathematical modeling was used to describe the relationships between variables, with polynomial models developed using the Taylor–MacLaurin series approximation (Goupy 2006). The models' quality was evaluated using coefficients of determination (*R* ^2^ and adjusted *R* ^2^), where *R* ^2^ ≥ 0.90 and adjusted *R* ^2^ ≥ 0.80 indicate a valid model (Goupy 2006; Joglekar and May 1987). The *p*‐value was used to determine the significance of coefficients, calculated from the ratio of the coefficient to its standard deviation (Goupy 2006).

### 2.3 Product Specification Document

The desired characteristics of the final product are outlined in Table 3.

**TABLE 3:** Product requirements document.

Characteristics	Standards/requirements
Physical characteristics	
Color	Close to that of the fresh product
Odor	Characteristics
Taste	Close to the fresh product
Chemical characteristics	
Moisture content (%)	< 10
Ash content (mg/100 g)	3.25
Vitamin, mineral, and polyphenol contents	Close to the fresh product
Microbiological characteristics	
Yeasts and molds (CFU/g)	< 1000
*E. coli* (UFC/g)	< 10
Salmonella	Absence

### 2.4 Optimization

The optimization process utilizes objective functions to maximize, minimize, or achieve target response values as specified in the design requirements (Table 4). This optimization was conducted using Design Expert 13 software.

**TABLE 4:** Optimization criteria.

Parameters/response	Target
Pineapple peel powder (A) (%)	—
Watermelon peel powder (B) (%)	—
Spearmint leaf powder (C) (%)	—
Moisture content	Targeted
Ash content	Minimize
Polyphenol content (mg/mL)	Maximize
Tannin content (mg/mL)	Minimize
Flavonoid content (mg/mL)	Maximize
Vitamin C (mg/mL)	Maximize
IC_50_ (mg/mL)	Maximize
Saponin content (mg/100 mL)	Minimize

### 2.5 Microbiological Analysis

The sample preparation, suspension, and decimal dilutions were performed according to the international standard ISO 6887‐1 (1999) (comité technique ISO/TC 34, 1999). The samples were analyzed immediately after sampling. Nine grams of sample were weighed into a sterile bag, and 90 mL of sterile peptone water was added. From this stock suspension, a series of successive decimal dilutions were prepared: 1 mL of solution was pipetted and introduced into a tube containing 9 mL of sterile peptone water at room temperature. One milliliter of this solution was then transferred to the next tube containing the same amount of peptone water (Table 5). The dilution process was continued to achieve the desired highest dilution. The pour plate method was used. One milliliter of each dilution was introduced into a sterile Petri dish, and molten culture medium was added at a temperature between 44°C and 47°C. The plates were allowed to solidify on a cold surface at room temperature before being incubated in an incubator.

**TABLE 5:** Microbiological analysis to be performed on the optimum tea.

Microorganisms to identify	Culture media	Incubation temperature (°C)	Incubation period (h)
Yeasts and molds	Sabouraud	25	48–72
Salmonella	SS	37	24
Total coliforms	MEB	37	24–48
*E. coli*s	MEB	44	24–48
Total aerobic bacteria count	PCA	30	48–72

The number of microorganisms per gram of powder is given by the following formula:

$$N=\mathrm{\Sigma C}/V\times \left(n_{1}+0.1n_{2}\right)\times d_{1}$$

where: *N* = number of microorganisms per gram of powder; ΣC = sum of colonies counted on all plates retained from two successive dilutions, with at least one plate containing 30 colonies; *n*_1_ = number of plates retained at the first dilution, *n*_2_ = number of plates retained at the second dilution, *V* = volume of inoculum applied to each plate, and *d*_1_ = dilution factor corresponding to the first dilution.

### 2.6 Animal Experimentation

The experiments and procedures were conducted in the animal house of the Laboratory of Food Technology, Food Science, and Nutrition at the University of Ngaoundere, Cameroon. The Institutional Animal Ethics Committee of the University of Ngaoundere, Cameroon, approved the study protocol. The animals were treated with care, and every effort was made to lessen their pain and suffering. The experiments were conducted on male albino Wistar rats weighing between 150 and 250 g. Two rat models were used: normoglycemic rats with blood glucose levels between 134 and 184 mg/dL and insulin‐resistant rats induced with dexamethasone (1 mg/kg) with blood glucose levels ≥ 190 mg/dL. The animals were fed a standard diet and had free access to water. The composition of the rat feed, on the basis of the standard diet by Hamlat et al. (2008) with some modifications, is presented in Table 6 (Hamlat et al. 2008).

**TABLE 6:** Composition of rat feed.

Constituent	Quantity (g)
Corn starch	590
Fish powder	200
Soybean oïl	50
Sucrose	50
Bone powder	50
Vitamin B complex	10
Cellulose	50
Total	1000

#### 2.6.1 Effect of Tea on Blood Glucose Levels in Normal Rats

Male rats were divided into groups of 5 and subjected to tests evaluating hypoglycemic activity and glucose tolerance. The rats fasted for 16 h before and during the experiment. Tea was infused in 200 mL of boiling water and administered via single gavage using a gastro‐esophageal tube. Blood samples were collected from a slight incision at the distal end of the tail and placed on glucose oxidase‐impregnated test strips. Blood glucose levels were measured using a glucometer.

##### 2.6.1.1 Hypoglycemic Activity Test

The procedure consisted of:

- A control group of 5 rats receiving distilled water via single gavage.
- Two test groups of 5 rats each receiving tea infusions at 300 and 500 mg/kg.

The administration volume was 10 mL/kg body weight. Blood glucose levels were measured at 0, 30, 60, 90, and 120 min.

##### 2.6.1.2 Glucose Tolerance Test

Twenty‐five male rats were fasted for 16 h and divided into 5 groups:

- Group 1 (Normal control): distilled water (10 mL/kg body weight)
- Group 2 (Positive control): metformin (0.3 mg/kg body weight)
- Group 3 (Negative control): 3 g/kg D‐glucose and distilled water (10 mL/kg body weight).
- Groups 4 and 5 (Test groups): tea extract at 300 and 500 mg/kg body weight.

Initial blood glucose levels were measured, followed by gavage with distilled water, metformin, or tea extract. Ninety minutes later, 3 g/kg D‐glucose was administered, and blood glucose levels were evaluated every 30 min for 2 h using a glucometer and test strips.

#### 2.6.2 Evaluation of the Protective Activity of Tea Extract on Dexamethasone‐Induced Insulin Resistance in Rats

Rats aged 10–12 weeks and weighing 150‐250 g were fasted for 16 h and divided into groups of 5 rats each. They were treated daily for 10 days as follows:

- Group 1 (Normal control) received distilled water (10 mL/kg, orally) and NaCl (1 mL/kg, subcutaneously).
- Group 2 (Negative control) received distilled water (10 mL/kg, orally) and dexamethasone (1 mg/kg, subcutaneously).
- Group 3 (Positive control): received metformin (40 mg/kg, orally) and dexamethasone (1 mg/kg, subcutaneously).
- Groups 4 and 5 received tea extract at 300 and 500 mg/kg, respectively, by gavage and dexamethasone (1 mg/kg, subcutaneously).

The tea was infused in boiling water for 5–10 min, cooled, and administered by gavage using a gastric tube at doses of 300 and 500 mg/kg body weight. Treatments were given daily at 8 am. Rat weights were recorded on the first day and every day thereafter (9 measurements). Weight gain or loss was calculated using the formula:

Weight gain/loss=Final weight−Initial weight

where initial weight: weight on the first day of experimentation; final weight: weight on the last day of experimentation.

#### 2.6.3 Rat Sacrifice and Organ Collection

At the end of the treatment period, the animals were fasted for 24 h with free access to water. The rats were then anesthetized via intraperitoneal injection of a ketamine (10 mg/mL) and diazepam (5 mg/mL) combination. The abdominal cavity was opened, and blood was collected through cardiac puncture. The blood was immediately centrifuged at 25,000 rpm for 10 min, and the collected serum was stored at −20°C for analysis of lipid parameters (cholesterol, HDL, LDL, triglycerides), transaminases (AST and ALT), and renal function parameters (urea, creatinine, and albumin). Subsequently, the liver, kidneys, heart, pancreas, and lungs were harvested, cleared of adipose tissue, rinsed in 0.9% NaCl solution, dried, and weighed to determine their relative mass.

#### 2.6.4 Biochemical Analysis

To evaluate the effects of the treatment on lipid profiles and liver and kidney function, biochemical parameters were measured, including total cholesterol (Naito 1984), HDL cholesterol (Gordon et al. 1977), triglycerides (Fossati and Prencipe 1982), LDL cholesterol is calculated using the Friedewald formula (Friedewald et al. 1972), alanine aminotransferase (ALAT) and aspartate aminotransferase (ASAT) (Reitman and Frankel 1957), and creatinine (Henry 1974). These parameters were analyzed using enzymatic and colorimetric methods with specific kits and protocols for each assay.

### 2.7 Statistical Analysis

For in vitro experiments, a D‐Optimal mixture design was generated using Design Expert software (version 13), which was also used for statistical mathematical modeling. For in vivo experiments, data analysis was performed using SPSS software, where the Kruskal–Wallis test was applied, followed by a Bonferroni post hoc test when significant differences were observed between groups. Results are presented as mean ± standard deviation (SD), and a P‐value ≤ 0.05 was considered statistically significant

## 3 Results and Discussion

### 3.1 Chemical Composition of Raw Materials

Table 7 shows the chemical properties of powders derived from *C. lanatus, A. comosus*, and *M. spicata* . The table provides an overview of the chemical characteristics of these raw materials.

**TABLE 7:** Chemical characteristics of the different raw materials.

Chemical constituents	pineapple peels	Watermelon peels	Spearmint leaves
Moisture content (%)	4.91407 ± 1.59^a^	6.08897 ± 0.4^a^	4.795 ± 1.67^a^
Dry matter (%)	95.0859 ± 1.59^a^	93.911 ± 0.45^a^	95.205 ± 1.67^a^
Ash content (%)	0.31836 ± 0.13^a^	0.281077 ± 0.14^b^	0.71 ± 0.07 ^c^
Vitamin C (mg/mL)	0.98 ± 0.00^a^	0.93 ± 0.00^a^	0.52 ± 0.00^b^
Flavonoid content (mg QE/100 g DM)	592.027 ± 0.00^a^	519.85 ± 0.00^b^	145.789 ± 0.00^c^
Tannin content (mg CAE/100 g DM)	5.40602 ± 1.286^a^	5.66445 ± 0.049^a^	8.44974 ± 2.302^a^
Polyphenol content (mg GAE/100 g DM)	6606.59 ± 0.00^a^	1305.09 ± 0.00^b^	369.289 ± 0.00^c^
Saponin content (mg)	1.5172 ± 0.106^a^	1.96 ± 0.305^a^	0.272 ± 0.00^b^
Total sugars	0.0918 ± 0.000^a^	0.0905 ± 0.000^a^	0.8710 ± 0.122^b^
Antioxidant activity (mg/mL)	126.505 ± 4.885^a^	133.568 ± 1.614^a^	8.380 ± 0.000^b^

*Note:* The data are expressed as mean ± standard deviation; Letters a, b and c denote significant differences (*p* < 0.05) between values in the same row.

From Table 7, it appears that watermelon peels have a moisture content of 6.088% ± 0.45%. This value is lower than that found by Neglo et al. (2021), which is 12.17%. Pineapple peels have a moisture content of 4.91407% ± 1.59%, a value lower than that found by Marquez Molina et al. (2023), which is 6.16% (Neglo et al. 2021; Marquez Molina et al. 2023). The moisture content of mint is 4.795% ± 1.67%. Indeed, moisture content is a crucial parameter in the storage of food products. A moisture content above 12% favors the development of microorganisms. In our study, the moisture content of the powder is below 10%, which could allow for good storage stability. Furthermore, high moisture content promotes fungal growth and could lead to changes in the nutritional composition of food products (Aryee et al. 2006).

The ash content of pineapple peels, watermelon peels, and mint leaves was found to be 0.31836 ± 0.13, 0.281077 ± 0.14, and 0.71 ± 0.07, respectively. The ash content is a measure of the total amount of minerals present within a food. It may vary depending on agricultural practices, plant maturity, and variety, which can affect mineral salt composition (Van Ryssen 2018).

According to the table, pineapple peels have a vitamin C content of 0.980 mg/mL, followed by watermelon peels. Pineapple peels also exhibit significantly higher flavonoid and polyphenol contents, with values of 592.027 ± 0.00 mg/100 g DM and 6606.59 ± 0.00 mg GAE/100 g DM, respectively, compared to watermelon peels and mint leaves. However, there is no significant difference in antioxidant activity between pineapple and watermelon peels, which both have significantly higher antioxidant activity than mint leaves. Notably, pineapple and watermelon peels contain 4 and 3.5 times more flavonoids than mint leaves, respectively. Additionally, both peels have significantly higher saponin content than mint leaves. In contrast, the difference in tannin content among pineapple peels, watermelon peels, and mint leaves is not significant.

Polyphenols, particularly flavonoids, have been shown to possess a range of biological activities that can contribute to the prevention and management of various diseases, including antioxidant activity (Hassanpour and Doroudi 2023), beneficial effects on cardiovascular health (Liu et al. 2024), anti‐inflammatory properties (Jameel et al. 2022), potential anticancer effects (de Luna et al. 2023), and neuroprotective effects (Amiri et al. 2024).

Flavonoids have been demonstrated to improve insulin sensitivity, reduce inflammation, and modulate glucose metabolism (Kim 2024). Saponins, particularly ginsenosides found in ginseng, have been shown to have anti‐diabetic effects by improving insulin sensitivity and glucose metabolism (Kim 2024). The antioxidant and anti‐inflammatory effects of both flavonoids and saponins may also help to mitigate oxidative stress and inflammation associated with diabetes (Le‐Le and Na 2025). Overall, the consumption of flavonoid‐ and saponin‐rich foods or supplements may be a useful adjunctive therapy for the management of diabetes.

### 3.2 Modeling the Impact of Raw Material Proportions on Tea Physicochemical Characteristics

The average raw results of physicochemical analyses of tea samples are presented in Table 8. Statistical mathematical modeling was applied to these results using Design Expert 13 software. The analysis matrix in Table 8 enabled the development of response models that relate individual factors to quadratic effects, providing insights into the relationships between the variables.

**TABLE 8:** Physicochemical characterization of tea formulations.

F	%AP	%PP	%MP	SC	FC	PC	TS	TC	AA	VIT C
F1	0.475	0.425	0.1	0.22 ± 0.01	858.89 ± 0.033	1134.06 ± 1.28	0.17 ± 0.00	0.038 ± 0.01	117.13 ± 63.90^b^	0.59 ± 0.056^b^
F2	0.5	0.35	0.15	0.22 ± 0.048	675.89 ± 0.14	1344.2 ± 2.76	0.10 ± 0.00	0.075 ± 0.00	162.22 ± 25.41^b^	0.435 ± 0.02^a^
F3	0.45	0.4	0.15	0.21 ± 0.030	667.89 ± 0.18	1884.06 ± 1.33	0.09 ± 0.00	0.049 ± 0.03	178.59 ± 1.50^c^	0.39 ± 0.05^a^
F4	0.43	0.43	0.14	0.23 ± 0.00	708.39 ± 0.08	1516.3 ± 1.25	0.11 ± 0.00	0.093 ± 0.00	179.44 ± 0.00^c^	0.62 ± 0.05^b^
F5	0.46	0.41	0.13	0.23 ± 0.00	770.89 ± 0.11	1610.51 ± 0.74	0.11 ± 0.01	0.098 ± 0.00	101.18 ± 2.67^a^	0.38 ± 0.05^a^
F6	0.45	0.45	0.1	0.23 ± 0.02	885.39 ± 0.157	1836.96 ± 1.22	0.10 ± 0.01	0.145 ± 0.03	95.23 ± 1.13^a^	0.44 ± 0.01^a^
F7	0.5	0.4	0.1	0.27 ± 0.00	980.89 ± 0.01	2521.74 ± 3.68	0.11 ± 0.00	0.092 ± 0.04	92.98 ± 1.20^a^	0.88 ± 0.02^c^
F8	0.4	0.45	0.15	0.26 ± 0.00	906.393 ± 0.05	2172.1 ± 0.64	0.09 ± 0.00	0.065 ± 0.03	92.99 ± 0.67^a^	0.88 ± 0.01^c^
F9	0.5	0.38	0.13	0.24 ± 0.01	940.89 ± 0.04	2076.09 ± 0.87	0.09 ± 0.00	0.065 ± 0.00	96.19 ± 0.45^a^	0.765 ± 0.02^c^

*Note:* The data are expressed as mean ± standard deviation; Letters a,b and c denote significant differences (*p* < 0.05) between values in the samerow.

Abbreviations: AA, Antioxidant activity (mg/mL); AP, *A. comosus* peel powder (g); F, formulation; FC, flavonoid content (mg/mL); MP, *M. spicata* powder (g); PC, Polyphenol content (mg/mL); PP, *C. lanatus* peel powder (g); SC, saponin content (mg); TC, tannin content (mg/mL); TS, total sugars (mg/mL); VIT C, vitamin C (meq A.A/mL).

### 3.3 Model Validation Criteria

The mathematical models are deemed valid on the basis of the following criteria: a coefficient of determination (*R* ^2^) of 0.9 or higher, and an adjusted *R* ^2^ value that is close to the *R* ^2^ value. The following Table 9 presents the *R* ^2^ and adjusted *R* ^2^ values for each model:

**TABLE 9:** Model validity characteristics.

Parameter	*R* ^2^	Adjusted *R* ^2^
Total polyphenols	0.9987	0.9974
Flavonoids	0.9221	0.8442
Antioxidant activity	0.9995	0.9991
Vitamin C	0.9533	0.9066
Saponin content	0.9272	0.8545
Tannin content	0.9884	0.9768

On the basis of Table 9, it is evident that all models are valid and suitable for exploratory analysis of the factors. For a factor to be considered significant, its *p*‐value must be less than 0.05 (*p* < 0.05). Consequently, only factors that meet this criterion will be taken into account.

The results of the analysis of variance (ANOVA) for the different responses are presented in Table 10.

**TABLE 10:** ANOVA results for different responses.

	A	B	C	AB	AC	BC
Total polyphenols	3491.22	957.943	2934.97	1669.85	−8455.46	2481.68
*p*	< 0.0001	< 0.0001	< 0.0001	< 0.0001	< 0.0001	0.0025
Flavonoïds	1487.62	1037.68	−412.743	1519.3	−734.043	1557.09
*p*	0.0096	0.0096	0.0096	0.0154	0.6248	0.2816
Antioxidant activity	−51.4612	4.91927	766.245	531.7	−278.687	−716.29
*p*	< 0.0001	< 0.0001	< 0.0001	< 0.0001	0.0002	< 0.0001
Vitamin C	1.90624	0.567971	−0.05711	2.60959	−3.647	2.41252
*p*	0.0310	0.0310	0.0310	0.0061	0.1158	0.2280
Saponin	0.351388	0.251612	0.054176	−0.248043	−0.132142	0.295869
*p*	0.0522	0.0522	0.0522	0.0079	0.5260	0.1566
Tannins	0.0421035	0.240087	−0.579297	−0.211724	1.07965	0.437244
*p*	0.0002	0.0002	0.0002	0.0008	0.0001	0.0044

Abbreviations: A, amount of pineapple; B, amount of watermelon; C, amount of mint.

### 3.4 Modeling the Impact of Factors on Physicochemical Properties of Extracts

The matrix presented in Table 11 allows for the derivation of models for the analyses performed on different responses using Design Expert. These models relate singular factors and interactions.

**TABLE 11:** Mathematical models for different responses.

Response	Model
Polyphenol content	+3491.22A+957.94B+2934.97C+1669.85AB‐8455.46 AC+2481.68BC
Tannin content	+0.0421A+0.2401B‐0.5793C‐0.2117AB+1.08 AC+0.4372BC
Flavonoid content	+884.55A+3957.02B+2606.31C+4056.10AB‐2271.93 AC‐7508.32BC
Antioxidant capacity	+2107.43A+2265.84B+998.50C+1681.09AB+252.32 AC‐1518.04BC
Vitamin C	+0.7427A+0.5544B+0.3836C+0.3329AB+0.3456 AC+0.1458BC

Abbreviations: A, amount of pineapple; B, amount of watermelon; C, amount of mint.

In the mathematical models presented above, the coefficients preceding each term illustrate the effect of the independent variables and the interaction between them. The parametric values represent the expected change in the response per unit change in the factor value when all other factors are held constant. The higher the parametric value, the more significant the weight of the variable. Furthermore, a synergistic effect is indicated by a positive sign, whereas a negative sign indicates antagonism (Pinela et al. 2018). The coefficients of the model are empirical in nature and do not have a direct physical or chemical interpretation. However, they are valuable for predicting the outcome of experimental conditions that have not been tested (Pinela et al. 2018).

### 3.5 Analysis of Interactions

**FIGURE 4:**
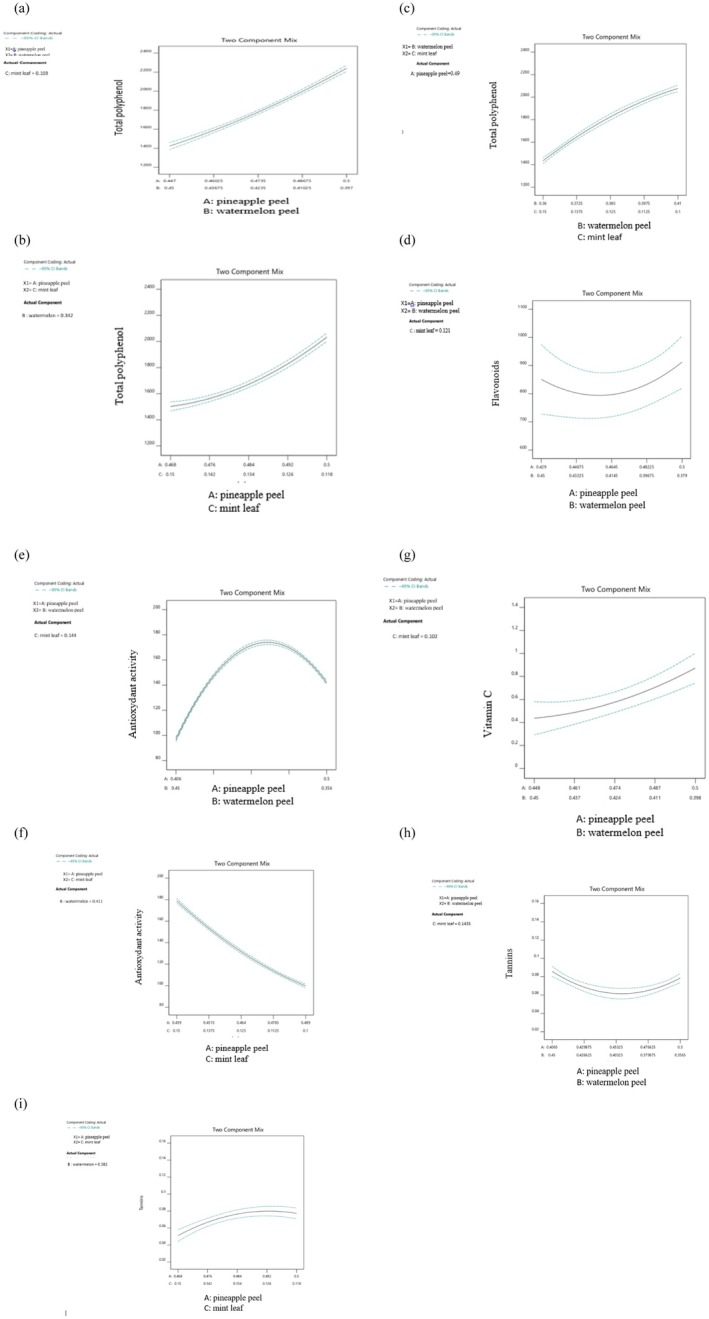
Interaction effects on various responses. (a): A/B interaction on polyphenol content, (b): A/C interaction on polyphenol content, (c): B/C interaction on polyphenol content, (d): A/B interaction on flavonoid content, (e): A/B interaction on antioxidant capacity, (f): A/C interaction on antioxidant capacity, (g): A/B interaction on vitamin C content, (h): A/B interaction on tannin content, (i): A/C interaction on tannin content.

Figure 4 presents the interaction effects between compounds A, B, and C on various responses, highlighting the interplay between these variables across multiple interactions (a to i).

Figure 4 (a) illustrates the relationship between polyphenol content and the quantities of pineapple peel and watermelon peel, revealing a significant increase in polyphenol content with the simultaneous increase in *C. lanatus* and *A. comosus* powders. This is likely due to the presence of phenolic compounds in both *C. lanatus* peels (Masih et al. 2021) and *A. comosus* peels (Uslu and Özcan 2024), which contributes to the higher polyphenol content in the tea samples. Notably, the main phenolic compounds found in pineapple peels include catechin (10.70–18.92 mg/100 g), gallic acid (10.18–17.25 mg/100 g), and ferulic acid (1.40–6.10 mg/100 g) (Uslu and Özcan 2024).

The relationship between polyphenol content and the quantities of pineapple peel (A) and mint leaf (C) is illustrated in Figure 4b. A significant increase in polyphenol content is observed with the simultaneous increase in *A. comosus* powder and *M. spicata* powder. The interaction between *A. comosus* and *M. spicata* contributes to a substantial increase in polyphenol content in the tea samples. This can be attributed to the presence of polyphenols in both mint leaves and *A. comosus* , as previously reported, suggesting that the combination of these two compounds in the sample enhances polyphenol content. The major phenolic compounds found in mint include rosmarinic acid, eriocitrin, luteolin 7‐O‐rutinoside, hesperidin, apigenin, pebrellin, and gardenin B (Safdar et al. 2022) (Brown et al. 2019).

Figure 4c illustrates the relationship between polyphenol content and the quantities of watermelon peel (B) and mint leaf (C). A significant increase in polyphenol content is observed when *A. comosus* powder increases and *M. spicata* powder decreases. This antagonistic effect may be due to the complex interactions between the bioactive compounds present in *A. comosus* and *M. spicata* . For instance, *A. comosus* peels are rich in phenolic compounds such as flavonoids and phenolic acids (Lourenço et al. 2021), which may contribute to the increased polyphenol content. On the other hand, *M. spicata* leaves contain a different profile of phenolic compounds, including rosmarinic acid and luteolin derivatives (Wani et al. 2022). The decrease in *M. spicata* powder may reduce the potential inhibitory effects of its compounds on the bioavailability or extractability of polyphenols from *A. comosus* , leading to an overall increase in polyphenol content. Further studies are needed to elucidate the exact mechanisms underlying this interaction effect.

The relationship between flavonoid content and the quantities of pineapple peel (A) and watermelon peel (B) is illustrated in Figure 4d. The optimal flavonoid content is achieved when either pineapple peel or watermelon peel is added in large quantities, but not both. This observation can be attributed to the complex interactions between the bioactive compounds present in both peels. For instance, pineapple peel contains enzymes like bromelain, a proteolytic enzyme that can degrade flavonoids and other bioactive compounds (Varilla et al. 2021). When both pineapple and watermelon peels are added simultaneously, the bromelain in pineapple peel may catalyze the degradation of flavonoids, leading to a decrease in flavonoid content. Furthermore, the combination of different flavonoid profiles in each peel may also contribute to the observed effect. Pineapple peel contains a unique profile of flavonoids, including quercetin and kaempferol derivatives (Bruce and Boateng 2025), whereas watermelon peel contains a different set of flavonoids, including naringenin and apigenin derivatives (Nissar et al. 2025). When both peels are added together, the different flavonoid profiles may interact and lead to a dilution effect, where the specific concentration of each flavonoid is reduced. However, when one peel is added in large quantities, and the other is decreased, a synergistic effect may occur, where the flavonoids from one extract are more stable and available because of the presence of other compounds (Senem et al. 2021). This synergistic effect may enhance the overall flavonoid content and contribute to the observed optimal flavonoid content when either pineapple or watermelon peel is added in large quantities.

Figure 4e shows the correlation between antioxidant capacity and the amounts of pineapple (A) and watermelon (B) peels. Antioxidant capacity increases with pineapple peel powder up to 180 mg/mL, then plateaus. This may be due to saturation of antioxidant compounds, including phenolic compounds, which scavenge free radicals and reduce oxidative stress because of their bioactive properties. Watermelon peel powder also plays a crucial role, interacting positively with pineapple peel powder up to a certain level. This synergistic effect may be due to the combination of different antioxidant compounds present in both peels, such as citrulline and other amino acids in watermelon peel (Gu et al. 2023). The interaction between these compounds can enhance the overall antioxidant capacity of the mixture. Maintaining an optimal balance between these two factors is essential to achieve maximum antioxidant capacity. This balance can be achieved by optimizing the ratio of pineapple peel to watermelon peel, as excessive amounts of either component may lead to a decrease in antioxidant activity. Further studies are needed to determine the optimal ratio and to elucidate the mechanisms underlying the synergistic effects observed.

Figure 4f shows the relationship between antioxidant capacity and the quantities of pineapple peel (A) and mint leaves (B). Antioxidant capacity decreases with increasing pineapple peel powder, and the simultaneous addition of both components reduces antioxidant capacity, likely due to interference from sugars and organic acids.

The evolution of vitamin C content as a function of the quantity of pineapple peel (A) and watermelon peel (B) is represented in Figure 4g. This figure shows a significant increase in vitamin C content with the simultaneous increase in the amount of *A. comosus* powder and *C. lanatus* powder. The interaction effect between *A. comosus* and *C. lanatus* contributes to a significant increase in vitamin C content in the tea samples. This could be attributed to the presence of vitamin C (ascorbic acid) in both *A. comosus* and *C. lanatus* , and the combination of these two compounds in the sample would therefore lead to an increase in vitamin C content in the mixture.”

Figure 4h shows the relationship between tannin content and the quantities of pineapple peel (A) and watermelon peel (B). Tannin content increases with pineapple peel powder, but the curve rises significantly when watermelon peel powder is decreased. This suggests that tannin content depends on the balance between these two factors. Some studies have shown that tannins interact with dietary proteins, affecting nutrient digestibility and bioavailability, and influencing nutrient absorption. (Cosme et al. 2025). The binding of tannins to proteins in watermelon peel may reduce their bioavailability.

The evolution of tannin content as a function of the quantity of pineapple peel (A) and mint (C) is represented in Figure 4i. The figure shows a significant increase in tannin content with the increase in the amount of pineapple peel powder and the decrease in the amount of mint powder. This finding is intriguing, as the characteristics of the raw materials indicate that mint leaves are significantly richer in tannins compared to pineapple peels. However, the observed result may be attributed to the extraction efficiency or the interaction between the compounds in the mixture, which could enhance the tannin content from pineapple peels. Indeed, it has been reported in the literature that pineapple peels contain tannins, particularly condensed tannins called proanthocyanidins (Mehraj et al. 2024). Further studies are needed to understand the underlying mechanisms and optimize the extraction conditions.

### 3.6 Optimization of Physicochemical Parameters

The optimization process aims to identify the ideal compromise for each response variable. This involves determining a suitable range that meets all the specified conditions and results in a tea that is physicochemically and nutritionally acceptable. The results of the optimization of the physicochemical parameters of the tea are presented in Table 12.

**TABLE 12:** Optimization of tea chemical parameters.

Parameters/response	Objectives	Results
Pineapple peel powder (A) (%)	—	43.39
Watermelon peel powder (B) (%)	—	41.06
Mint powder (C) (%)	—	15.16
Flavonoid content (mg/mL)	Maximize	813.986
Tannin content (mg/mL)	Minimize	0.056
Polyphenol content (mg/mL)	Maximize	2019.35
Vitamin C (meqgA. As/mL)	Maximize	0.735
Antioxidant activity (mg/mL)	Maximize	128.86

### 3.7 Microbiological Analysis of the Tea Sample

The results of the microbiological analysis reveal that the microbial load varies according to the type of microorganism and the sample analyzed. The total flora is predominant, followed by total coliforms and yeasts, with respective values of 1.5 × 10^4^ CFU/g, 2.4 × 10^3^ CFU/g, and 1.2 × 10^2^ CFU/g for the tea sample. The level of contamination is within the acceptable limits according to our specifications. The microbial load of the optimal tea formulation is presented in Table 13.

**TABLE 13:** Microbial load of tea samples (CFU/g).

Microorganisms/group	Tea	Standard
Total aerobic flora (CFU/g)	1.5 × 10^4^	**10** ^**5**^
Yeasts (fungal) (UFC/g)	1.2 × 10^2^	
Total coliforms (UFC/g)	2.4 × 10^3^	**10** ^**3**^
*Escherichia coli* (UFC/g)	Absent	

*Note:* The bold values in table 13 represent the microbiological standards or limits for the tea product.

### 3.8 In Vivo Evaluation of the Biological Activities of Watermelon Peel, Pineapple Peel, and Mint Leaf Tea

This part of the study investigated the hypoglycemic and anti‐hyperglycemic effects of two doses of tea in normal and insulin‐resistant rats. Blood glucose levels were measured using a glucometer and OneTouch Ultra glucose test strips, with normal levels defined as 134–184 mg/dL. Rats were classified as hypoglycemic (≤ 134 mg/dL) or hyperglycemic (≥ 184 mg/dL) on the basis of their blood glucose levels.

#### 3.8.1 Effect of Tea on Blood Glucose Levels in Normal Rats

##### 3.8.1.1 Hypoglycemic Activity Test

**FIGURE 5:**
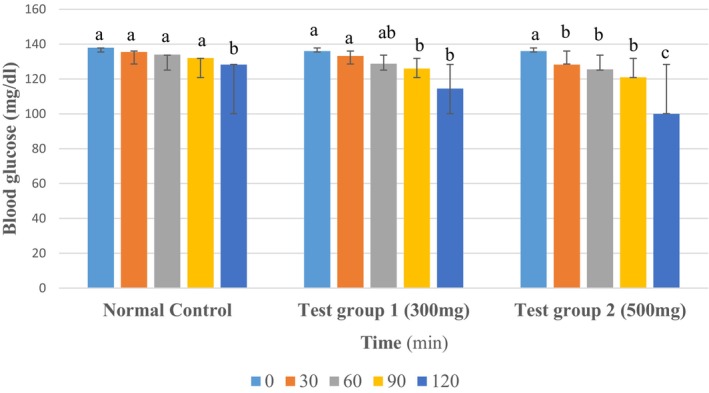
Hypoglycemic test in normal rats. The data are expressed as mean ± standard deviation. Letters a, b, and c denote significant differences (*p* < 0.05) between values in the same batches.

The hypoglycemic activity of tea was evaluated in normal rats using two doses (300 and 500 mg/kg). Figure 5 presents the results of blood glucose levels (in mg/dl) obtained at different times after administration of the extracts. The values are represented as mean ± standard deviation.

The results showed a decrease in blood glucose levels after 30 min in all treated groups. After 120 min, significant decreases in blood glucose levels were observed in the CT300 and CT500 groups compared to the initial time point (T0 = 0 h). Furthermore, significant differences were observed between the groups gavaged with extracts and the normal control group that received distilled water (*p* < 0.05).

The comparison was performed with respect to the initial time point (T0 = 0 h) and also with respect to the untreated group (negative control).

In the normal control group, blood glucose levels remained relatively stable with a slight decrease, likely due to normal fluctuations. In healthy individuals, blood glucose is a tightly regulated variable, maintained within narrow limits, both during fasting and postprandial periods (Scheen and Paquot 2006). The decrease in blood glucose over time observed in untreated normal subjects has also been reported by Vitoriano et al. (2011), who attributed it to the prolonged fasting conditions experienced by the rats during experimentation (Vitoriano et al. 2011). During prolonged fasting, energy expenditure is almost exclusively met by the oxidation of endogenous lipids, either directly via beta‐oxidation of fatty acids or indirectly via conversion of fatty acids to ketone bodies. Without exogenous carbohydrate intake, blood glucose levels initially decrease but then stabilize at low physiological values (3–4 mmol/L) because of hepatic gluconeogenesis (Luc 2017). However, in the groups receiving different doses of tea, the decrease in blood glucose levels from 60 min onwards was significant, demonstrating the hypoglycemic effect of tea. The decrease in blood glucose levels observed from 30 min onwards in rats gavaged with tea made from watermelon peels, pineapple, and mint leaves at doses of 300 and 500 mg/kg may be attributed to the presence of bioactive compounds in these matrices, such as flavonoids (notably quercetin), polyphenols, and phenolic acids (Neglo et al. 2021). Indeed, these compounds can improve insulin sensitivity by activating intracellular signaling pathways such as the AMPK (AMP‐activated protein kinase) pathway or increasing the expression of glucose transporters (GLUT4) in peripheral tissues (Moloto et al. 2020). They can also reduce hepatic glucose production by inhibiting enzymes involved in gluconeogenesis (Tran et al. 2020).

##### 3.8.1.2 Oral Glucose Tolerance Test (OGTT)

**FIGURE 6:**
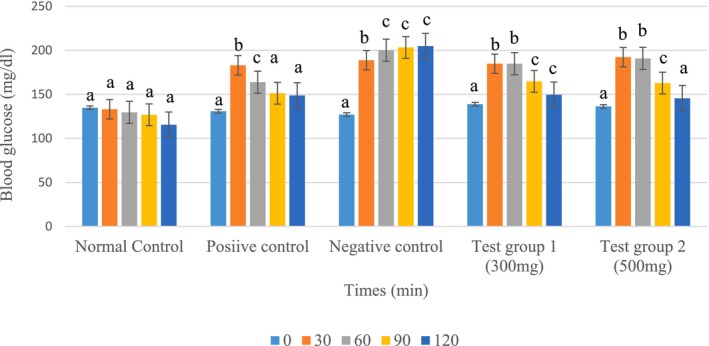
Oral glucose tolerance test. The data are expressed as mean ± standard deviation. Letters a, b, and c denote significant differences (*p* < 0.05) between values in the same batches.

The study of the anti‐hyperglycemic activity (Figure 6) of the two tea doses showed that the peak blood glucose level was reached 30 min after administration of the tea extracts and glucose. A decrease in blood glucose was observed 90 min after the start of the experiment in all groups. After 120 min, CT300 and CT500 showed significant decreases in blood glucose compared to the negative control group.

The results of the study demonstrate that the tea extracts exhibited significant anti‐hyperglycemic activity in normoglycemic rats, with a notable hypoglycemic effect observed after 90 min (*p* < 0.05). This finding is consistent with previous studies that have shown that polyphenols and flavonoids present in tea extracts can act as insulin sensitizers and inhibitors of carbohydrate‐digesting enzymes, such as alpha‐amylase and alpha‐glucosidase (Binti Ismail et al. 2018; Lam et al. 2024). The rapid absorption of glucose in the gastrointestinal tract following administration of the hyperglycemic solution leads to a transient increase in blood glucose, which is observed at T30 (Dimitriadis et al. 2021). However, the pancreas responds to this increase in blood glucose by secreting insulin, which promotes glucose uptake by peripheral tissues, including muscle, liver, and adipose tissue. The delayed effect of insulin may explain the progressive decrease in blood glucose from T90 (Dimitriadis et al. 2021). The tea extract's bioactive compounds, including polyphenols and flavonoids, may contribute to the observed anti‐hyperglycemic effect by slowing down glucose absorption and improving glucose utilization (Luo et al. 2024). This is supported by studies that have shown that polyphenols and flavonoids can inhibit alpha‐amylase and alpha‐glucosidase activity, thereby reducing glucose absorption and improving insulin sensitivity (Binti Ismail et al. 2018; Lam et al. 2024). Notably, the results show that both tea doses (300 and 500 mg/kg bw) exhibited hypoglycemic and anti‐hyperglycemic activity in normal rats, with a predominance for the 500 mg/kg bw dose. This suggests that the higher dose may be more effective in modulating glucose metabolism and improving insulin sensitivity. Further studies are needed to confirm this finding and to elucidate the underlying mechanisms.

#### 3.8.2 Evaluation of the Protective Effect of Tea Extract on Glycemia in Dexamethasone‐Induced Diabetic Rats

##### 3.8.2.1 Glycemic Changes in Response to Dexamethasone‐Induced Insulin Resistance

**FIGURE 7:**
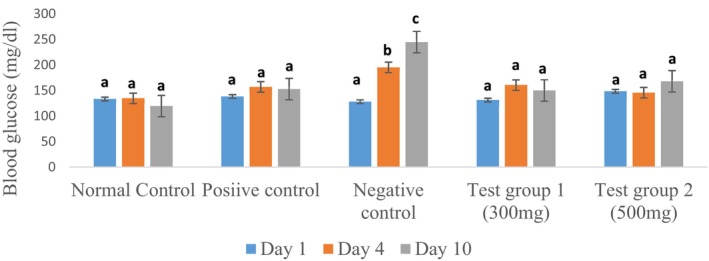
Change in blood glucose levels during the induction of insulin resistance. The data are expressed as mean ± standard deviation. Letters a, b, and c denote significant differences (*p* < 0.05) between values in the same batches.

Blood glucose levels were monitored throughout the induction of insulin resistance by dexamethasone on days 1, 4, and 10. The graph in Figure 7 illustrates the changes in blood glucose levels over time.

Figure 7 shows that blood glucose levels remain stable in the normal control group, whereas in rats receiving tea extracts and metformin along with dexamethasone, there is a non‐significant increase in blood glucose levels on day 4, which is subsequently stabilized by the tea extract at a dose of 300 mg and metformin on day 10. In contrast, at a dose of 500 mg, a slight increase in blood glucose levels is still observed on day 10. In the negative control group, which received only dexamethasone and distilled water, blood glucose levels increased significantly from day 4 to day 10. Notably, blood glucose levels exceeded 200 mg/dL, indicating the onset of diabetes in these untreated rats compared to baseline levels. By day 10, the rats in the negative control group had already developed overt diabetes. These findings suggest that tea extracts and metformin have a protective effect against dexamethasone‐induced hyperglycemia. The stabilization of blood glucose levels in rats receiving tea extracts and metformin may be attributed to their ability to improve insulin sensitivity and reduce hepatic glucose production. Dexamethasone is known to induce insulin resistance by reducing insulin sensitivity and increasing hepatic glucose production (Beaupere et al. 2021). Tea extracts, particularly flavonoids and polyphenols, have been shown to improve insulin sensitivity and reduce hepatic glucose production (Hassanpour and Doroudi 2023; Hanhineva et al. 2010). Metformin, a well‐established medication for type 2 diabetes, acts by reducing hepatic glucose production and improving insulin sensitivity (Foretz et al. 2021). Notably, the results also indicate that the 300 mg dose of tea extract is more effective than the 500 mg dose in stabilizing blood glucose levels. This may be due to the presence of different bioactive compounds in tea extracts at varying doses or distinct mechanisms of action. In conclusion, these findings suggest that tea extracts have a protective effect against dexamethasone‐induced hyperglycemia, with the 300 mg dose of tea extract being more effective than the 500 mg dose.

##### 3.8.2.2 Effect of Tea Consumption on Animal Weight and Relative Organ Mass

The impact of watermelon and pineapple peel tea consumption on body weight and relative organ mass in albino rats is presented in Table 14, which shows the weight and relative mass of various organs in rats administered different doses of the tea

**TABLE 14:** Relative organ mass and body weight of rats fed tea at different doses.

	Normal control	Negative control	Positive control	Test group 1 (300 mg)	Test group 2 (500 mg)
G/P (g)	19.3 ± 17.21^a^	33.33 ± 26.27^b^	32.33 ± 9.23^b^	25.66 ± 6.65^ab^	19.0 ± 9.04^a^
Liver	3.59 ± 0.65^a^	6.65 ± 2.01^b^	6.47 ± 1.0^b^	6.45 ± 0.61^b^	6.07 ± 0.65^b^
Kidney	0.94 ± 0.15^a^	1.24 ± 0.22^a^	0.99 ± 0.23^a^	1.13 ± 0.16^a^	1.25 ± 0.26^a^
Fat	1.16 ± 0.44^a^	1.41 ± 0.66^a^	1.29 ± 0.14^a^	1.16 ± 0.58^a^	0.98 ± 0.16^a^

*Note:* The data are expressed as mean ± standard deviation. Letters a and b denote significant differences (*p* < 0.05) between values in the same row.

Abbreviations: G, weight gain; P, initial body weight.

The study also investigated the effects of tea consumption on body weight and organ mass in rats treated with dexamethasone. According to previous studies, changes in relative organ mass after dexamethasone consumption can indicate toxicity (Chen et al. 2019; Lu et al. 2023). The results in Table 14 showed a variation in body weight among the different animal groups. Notably, rats fed with tea consumed more food compared to the normal control group, suggesting that tea consumption may stimulate appetite and protect rats against the effects of dexamethasone. Although a non‐significant decrease in body weight was observed in all tea‐treated groups, this decrease was dose‐dependent and could be attributed to the presence of significant amounts of phenolic compounds in tea, which have been shown to inhibit nutrient absorption (Matsumura et al. 2023). Regarding organ mass, our results showed no significant differences (*p* > 0.05) in relative liver, kidney, and fat mass between the different tea doses used, suggesting that tea consumption has no influence on relative organ mass. Importantly, this study highlights two potential activities of tea: hepatoprotective and non‐toxic activities. These findings are consistent with recent studies that have confirmed the potential benefits of tea consumption on health.

##### 3.8.2.3 Effect of Consuming Tea Made From the Peels of Watermelon, Pineapple, and Mint Leaves on the Lipid Profile of Rats

**FIGURE 8:**
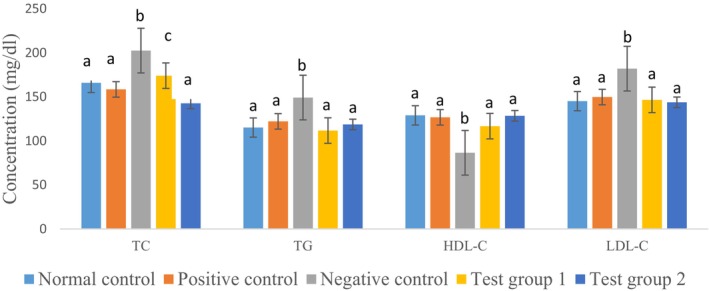
Effects of Tea Consumption on Serum Lipid Profiles in Rats. The data are expressed as mean ± standard deviation. Letters a, b, and c denote significant differences (*p* < 0.05) between values in the same batches. HDL‐C, HDL cholesterol; LDL‐C, LDL cholesterol; TC, total cholesterol; TG, triglycerides.

Figure 8 illustrates the effects of tea made from watermelon peel, pineapple peel, and mint leaves on the lipid profile, specifically total cholesterol, HDL cholesterol, LDL cholesterol, and serum triglycerides. A significant difference (*p* < 0.05) was observed between insulin‐resistant rats not treated and normal rats.

It is well‐established that any factor influencing glucose metabolism under different conditions can also impact lipid metabolism (Parhofer 2015). Additionally, it has been shown that triglyceride accumulation is more pronounced in diabetes (Alexopoulos et al. 2019). The analyzed parameters (total cholesterol, triglycerides, LDL cholesterol, and HDL cholesterol) reflect the metabolic and lipid status of diabetic rats and allow for the evaluation of the impact of dexamethasone and the protective effects of metformin and tea at different doses.

The results of this study show a normal lipid profile in the normal control group, with normal levels of total cholesterol (TC), triglycerides (TG), LDL cholesterol (LDL‐C), and HDL cholesterol (HDL‐C), indicating a balanced lipid metabolism. In contrast, insulin‐resistant rats exhibit a marked increase in triglycerides, total cholesterol, and LDL cholesterol, accompanied by a decrease in HDL cholesterol. These disturbances indicate a dexamethasone‐induced dyslipidemia, which leads to insulin resistance and altered lipid metabolism (Batista et al. 2024). Indeed, dexamethasone induces a diabetic state by increasing insulin resistance, which disrupts lipid metabolism. Hyperglycemia promotes lipolysis and the release of free fatty acids, which are then converted to triglycerides and LDL cholesterol in the liver, exacerbating dyslipidemia (Batista et al. 2024). In the positive control group (treated with metformin), metformin significantly reduces TC, TG, and LDL‐C levels while increasing HDL‐C levels. This confirms its beneficial effects on lipid metabolism by improving insulin sensitivity and reducing hepatic lipogenesis. Concerning the group receiving tea at a dose of 300 mg/kg, we observed that at this dose, tea reduces TC, TG, and LDL‐C levels compared to the negative control group. However, the levels of these markers remain slightly higher than those in the positive control group. HDL‐C levels increase, indicating a partial improvement in lipid profile. In contrast, the group receiving tea at a dose of 500 mg/kg shows an even more pronounced reduction in TC, TG, and LDL‐C levels, reaching levels similar to those in the positive control group. HDL‐C levels are significantly increased, suggesting better efficacy of tea at this dose.

##### 3.8.2.4 Effect of Watermelon Peel, Pineapple Peel, and Mint Leaf Consumption on Transaminase Activity (ALT and AST)

**FIGURE 9:**
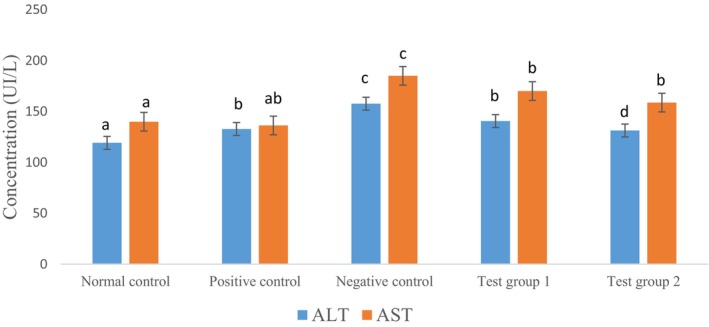
Effect of Tea on ALT and AST Activity. The data are expressed as mean ± standard deviation. Letters a, b, c, and d denote significant differences (*p* < 0.05) between values on the same batches.

Figure 9 shows the impact of tea consumption on the activity of alanine transaminase (ALT) and aspartate transaminase (AST), two key markers of hepatic injury.

Alanine aminotransferase (ALT) and aspartate aminotransferase (AST) are essential indicators of liver damage and allow monitoring of necrosis and inflammation. Normally, these enzymes are present in large quantities in the liver, with ALT mainly found in the hepatic cytosol and AST found in the liver, heart, and skeletal muscles. During liver necrosis, an increase in transaminases is generally observed, with a ratio of ALT/AST greater than 1 (Rej 1989).

According to Figure 9, the administration of dexamethasone to rats resulted in a significant increase (*p* ≤ 0.001) in ALT activity in animals compared to the normal control. However, treatment with different doses of tea and metformin significantly reduced (*p* ≤ 0.001) ALT activity in animals compared to the negative control. The normal control group showed the lowest levels of ALT, indicating an absence of liver stress. In contrast, the negative control group showed a significant increase in ALT levels, reflecting severe liver stress caused by the diabetic state induced by dexamethasone. Treatment with tea at a dose of 300 mg/kg (CT300) resulted in a significant reduction in ALT levels compared to the negative control group, indicating a certain level of protection against liver damage related to diabetes. Furthermore, treatment with tea at a dose of 500 mg/kg (CT500) resulted in even lower ALT levels, similar to those observed in the positive control group (CP) treated with metformin. This suggests a dose‐dependent effect of tea, with better liver protection at 500 mg/kg. The bioactive compounds present in tea, such as flavonoids (quercetin, catechins, etc.), may reduce oxidative stress and improve liver function, as shown by the dose‐dependent reduction in ALT.

The results of our study also showed that dexamethasone induced a significant increase (*p* ≤ 0.001) in AST activity in untreated animals compared to the normal control. However, treatment with tea and metformin at different doses significantly reduced (*p* ≤ 0.001) AST activity in animals compared to the negative control. The increase in AST activity may be due to liver damage caused by dexamethasone, which can lead to the release of enzymes into the blood (Danboyi et al. 2022). Treatment with tea reduced AST levels, suggesting that tea protects hepatocytes against free radical attacks and decreases the leakage of intracellular enzymes by preserving the plasma membrane.

##### 3.8.2.5 Effect of Tea Consumption on Renal Function Markers: Urea, Creatinine, and Albumin

Urea and creatinine are biomarkers of renal function. According to Table 15, induction of type 2 diabetes by dexamethasone significantly increased (*p* ≤ 0.001) serum urea and creatinine levels compared to the normal control. This increase may be attributed to nephrotoxicity caused by diabetes and dexamethasone, resulting from impaired glomerular filtration and increased protein catabolism (Cohen et al. 2022) for urea and increased degradation of protein compounds to amino acids and then to creatinine (Ávila et al. 2025) for creatinine. In contrast, treatment with tea at different doses and metformin significantly decreased (*p* ≤ 0.001) both urea and creatinine concentrations compared to the negative control. The antioxidant effect of tea may protect the kidneys against the adverse effects of dexamethasone by enhancing cellular defense processes against the cytotoxic effects of free radicals.

**TABLE 15:** Impact of tea consumption on kidney damage parameters.

	Normal control	Positive control	Negative control	Test group 1	Test group 2
Urea (g/L)	0.162^a^	0.195^ab^	0.3325^c^	0.2445^d^	0.19075^a^
Creatinine (mg/L)	4.75^a^	5.95^a^	16.225^b^	5.75^a^	4^a^
Albumin (g/L)	37.35^a^	37.15^a^	56.05^b^	39.875^a^	45.35^c^

*Note:* The data are expressed as mean ± standard deviation; Letters a, b, c and d denote significant differences (*p* < 0.05) between values in the same row.

Regarding albumin concentration, the negative control group had significantly higher levels compared to the normal control, indicating an abnormal liver function response in the diabetic state. However, treatment with tea (300 mg) and metformin resulted in albumin levels similar to those of the normal control. Notably, the tea‐treated group (300 mg) exhibited albumin levels similar to those observed in the positive control group, suggesting that this tea dose possesses hypoglycemic and hepatoprotective properties comparable to a reference treatment.

## 4 Conclusion

The general objective of this study was to evaluate the hypoglycemic and anti‐hyperglycemic effects of tea consumption made from pineapple peels, watermelon peels, and mint on the metabolism of Wistar albino rats. The results showed that the plant‐based raw materials were rich in bioactive compounds, particularly phenolic compounds (flavonoids, tannins, saponins, and polyphenols). The optimized tea, rich in antioxidants such as flavonoids and polyphenols, exhibited hypoglycemic and anti‐hyperglycemic effects in normal rats. Efficacy was observed after 60 min at doses of 300 mg/kg and 500 mg/kg body weight. In rats rendered diabetic with dexamethasone, in addition to the significant decrease in blood glucose peaks between 60 and 90 min in the test groups receiving tea and the reference medication, we also noted a significant decrease in total cholesterol, LDL cholesterol, and triglyceride levels. Similarly, tea consumption led to a significant increase in HDL cholesterol levels. During this experiment, liver and kidney functions were protected by the tea doses, as we observed a decrease in AST and ALT transaminase levels, as well as a decrease in urea, creatinine, and albumin levels in all treated groups. The dose of 500 mg/kg body weight was the most effective. These optimal characteristics, including the high levels of flavonoids and polyphenols, are crucial for the tea's therapeutic potential in managing diabetes. Given the promising results, future studies should focus on translational research, including clinical trials, to explore the potential of this tea as a complementary therapy for diabetes management. Additionally, further research could investigate the specific mechanisms of action and the optimal dosage for human consumption.

## Author Contributions

**Irwin Leoda Begnone:** conceptualization (equal), data curation (lead), formal analysis (lead), investigation (lead), methodology (equal), writing – original draft (equal). **Laurette Blandine Mezajoug Kenfack:** conceptualization (equal), methodology (equal), resources (equal), supervision (equal), validation (equal), writing – review and editing (supporting). **Rosane Matsinkou Soh:** conceptualization (equal), methodology (equal), resources (equal), supervision (equal), validation (equal), visualization (equal), writing – original draft (equal), writing – review and editing (lead).

## Conflicts of Interest

The authors declare no conflicts of interest.

## Data Availability

The data that support the findings of this study are available from the corresponding author upon reasonable request.
